# Nuclear EGFR impairs ASPP2-p53 complex-induced apoptosis by inducing SOS1 expression in hepatocellular carcinoma

**DOI:** 10.18632/oncotarget.3757

**Published:** 2015-04-27

**Authors:** Kai Liu, Tao Jiang, Yabo Ouyang, Ying Shi, Yunjin Zang, Ning Li, Shichun Lu, Dexi Chen

**Affiliations:** ^1^ Beijing You'an Hospital, Capital Medical University, Beijing, 100069, China; ^2^ Beijing Institute of Hepatology, Beijing, 100069, China

**Keywords:** ASPP2, p53, SOS1, EGFR, apoptosis

## Abstract

ASPP2 can bind to p53 and enhance the apoptotic capabilities of p53 by guiding it to the promoters of pro-apoptotic genes. Here, ASPP2 overexpression for 24 hours transiently induced apoptosis in hepatoma cells by enhancing the transactivation of p53 on pro-apoptotic gene promoters. However, long-term ASPP2 overexpression (more than 48 hours) failed to induce apoptosis because p53 was released from the pro-apoptotic gene promoters. In non-apoptotic cells, nuclear EGFR induced SOS1 expression by directly binding to the *SOS1* promoter. SOS1 activated the HRAS/PI3K/AKT pathway and resulted in nuclear translocation of p-AKT and Bcl-2. The interaction between p-AKT and ASPP2 facilitates Bcl-2 binding to p53, which releases p53 from the pro-apoptotic gene promoters. The *in vivo* assay demonstrated that EGFR/SOS1-promoted growth of nuclear p-AKT^+^, Bcl-2^+^ cells results in the resistance of hepatoma cells to ASPP2-p53 complex-induced apoptosis and that blocking nuclear translocation of EGFR dramatically improves and enhances the pro-apoptotic function of ASPP2. Finally, the activation of the HRAS/PI3K/AKT pathway by EGFR-induced SOS1 also inhibits cisplatin-induced apoptosis, suggesting a common apoptosis-evasion mechanism in hepatoma cells. Because evasion of apoptosis contributes to treatment resistance in hepatoma, our results also support further investigation of combined therapeutic blockade of EGFR and SOS1.

## INTRODUCTION

Apoptosis is involved in the regulation of many physiological and pathological processes in cells [1]. Insufficient apoptotic cell death promotes tumorigenesis [2]. Moreover, evasion of apoptosis is one of the hallmarks of tumors that result in treatment resistance [2]. The discovery of apoptosis escape mechanisms has great potential to help guide the design of novel therapeutic strategies for human cancer [2]. Hepatocellular carcinoma (HCC) represents one of the most difficult cancers to treat [3], and the current therapeutic strategies designed to induce apoptosis are still not effective enough to completely cure HCC [4].

Apoptosis-stimulating protein of p53-2 (ASPP2) is characterized by the presence of a proline-rich region, an SH3 domain, and ankyrin repeats [5, 6] and has previously been identified as an interacting partner for Bcl-2 and p53 [7]. ASPP2 binds to p53 through its C-terminus to stimulate the transactivation function of p53 on the promoters of pro-apoptotic genes, e.g., *PUMA* (p53 upregulated modulator of apoptosis). ASPP2 is a haploinsufficient tumor suppressor, and ASPP2^+/−^ mice are prone to developing cancer [8]. Our previous study suggests a potential role of ASPP2 in inhibiting HCC by promoting C/EBP Homologous Protein (CHOP)-mediated autophagic apoptosis [9].

A clear link has been established between the PI3K/AKT pathway and the pathogenesis of HCC [10]. AKT is a key player in the PI3K pathway [11]. Activation of AKT might predict poor prognosis in HCC [12]. Activated HRAS can activate p110 PI3K in a p85 PI3K-dependent and -independent manner [13]. Constitutive activation of the PI3K/AKT signaling pathway often enables cells to proliferate in an uncontrolled manner.

The tumor suppressor p53 was proposed to activate a cell cycle check and to induce apoptosis, whereas the proto-oncogene Bcl-2 functions as an inhibitor of cell death [14]. p53 inhibits Bcl-2 via promoting its antagonists or via repressing Bcl-2 transcription in some settings [14]. In mitochondria, p53 directly binds to Bcl-2 via its DNA-binding domain and then induces apoptosis [15–17].

To our knowledge, this is the first report demonstrating that nuclear EGFR can induce Son of Sevenless 1 (SOS1) expression by directly binding to the *SOS1* promoter; SOS1 then impairs ASPP2-induced apoptosis in hepatoma cells. We believe that our data can explain some of the cases where p53 is normal but apoptosis cannot be successfully induced in cancer cells.

## RESULTS

### Long-term ASPP2 overexpression fails to induce apoptosis in hepatoma cells

**Figure 1:**
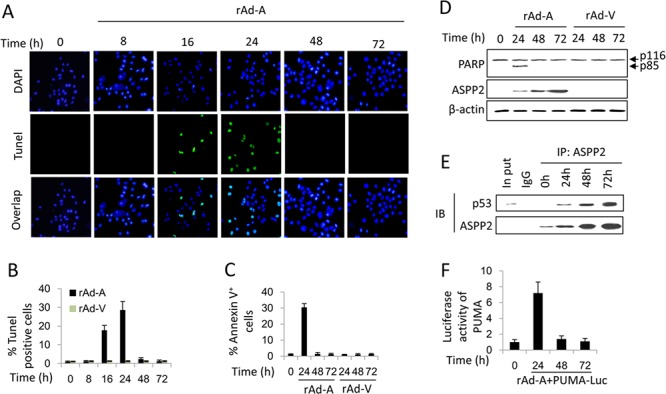
ASPP2-induced apoptosis is impaired in hepatoma cells **A.** TUNEL assay was used to detect the effect of rAd-ASPP2 (rAd-A) infection-induced ASPP2 overexpression on apoptosis induction in HepG2 cells at 8, 16, 24, 48, and 72 hours. **B.** Levels of apoptotic cells in (A) are the mean ± SEM of triplicates. **C.** Annexin V/PI assay was used to detect apoptosis (Annexin V^+^ cells). Values are the mean ± SEM of triplicates. **D.** Immunoblot assay was used to detect apoptosis in HepG2 cells infected with rAd-A or rAd-Vector (rAd-V) for the indicated times. Arrow indicates cleaved PARP fragment. **E.** CO-IP assay was used to detect the formation of ASPP2-p53 complex in HepG2 cells infected with rAd-A for the indicated times. **F.** Luciferase activity of the *PUMA* promoter-reporter constructs after transfection into HepG2 cells is shown. Values are the mean ± SEM of triplicates.

HepG2 cells were infected by rAd-ASPP2 for 8, 16, 24, 48 and 72 hours. TUNEL, immunoblotting and Annexin V/PI assays showed that in HepG2 cells, ASPP2 overexpression-induced apoptosis could be detected at 16 and 24 hours but not at 48 and 72 hours (Figure 1A, 1B, 1C and 1D). ASPP2 overexpression also induced apoptosis at 24 hours in PHCs but not at 48 and 72 hours (Supplementary Figures 1). However, ASPP2 levels at 48 or 72 hours were higher than that at 24 hours in HepG2 and PHCs cells (Figures 1D and S1). Recombinant adenovirus-Vector (rAd-Vector) had no effect on apoptosis (Figures 1B, 1C and 1D).

PCR array and immunoblot assays showed that the mRNA and protein levels of seven p53-regulated pro-apoptotic genes (*DR5*, *APAF1*, *BAX*, *Caspase 9*, *El24*, *TRAIL*, and *PUMA*) were increased at 16 and 24 hours (Figure S2A and S2B). Knockdown of the seven genes via siRNAs revealed that the expressions of PUMA, BAX and caspase 9 were mainly involved in ASPP2-induced apoptosis at 24 hours (Figure S2C). Although the ASPP2-p53 complex could be detected up to 72 hours by a CO-IP assay, a luciferase assay showed that ASPP2 promoted PUMA expression only at 24 hours but not at 48 and 72 hours (Figures 1E and 1F). Thus, ASPP2 overexpression for 24 hours transiently induces apoptosis by enhancing the transactivation activation of p53. However, the effect of long-term ASPP2 overexpression (more than 48 hours) on inducing apoptosis is impaired in hepatoma cells.

### Nuclear p-AKT and Bcl-2 impair ASPP2-induced apoptosis by interfering with the interaction between p53 and the promoter region of pro-apoptotic genes

**Figure 2:**
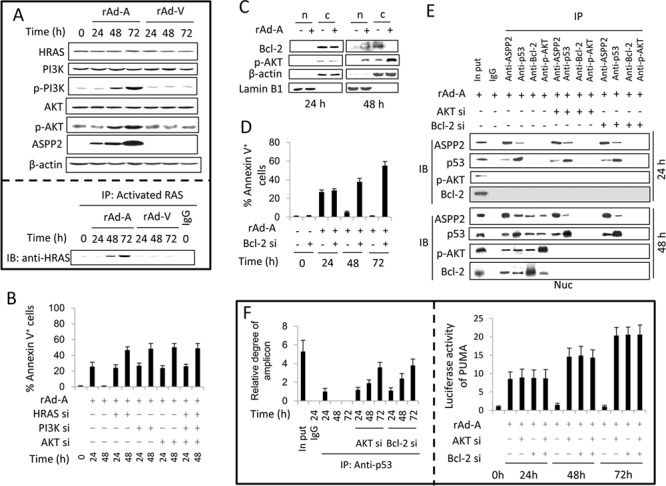
Activation of the HRAS/PI3K/AKT pathway inhibits ASPP2-induced apoptosis (**A.** upper panel) Immunoblot assay was used to detect the activation of the HRAS/PI3K/AKT pathway in HepG2 cells infected with rAd-ASPP2 (rAd-A) and rAd-Vector (rAd-V) for the indicated times. (A, lower panel) Anti-RAS-GTP antibody (activated RAS) was used to immunoprecipitate total activated RAS, and anti-HRAS was used to detect HRAS levels in total activated RAS. **B** and **D.** Annexin V/PI assay was used to detect ASPP2-induced apoptotic cells (Annexin V^+^ cells) after transfection with siRNA for HRAS, PI3K, AKT, and Bcl-2. Values are the mean ± SEM of triplicates. **C.** HepG2 cells were infected with rAd-A for 24 and 48 hours. Nuclei (n) and nuclei-free cytoplasm (c) were isolated and immunoblot assay was used to detect Bcl-2 and p-AKT levels in isolated nuclei and cytoplasm fractions. **E.** rAd-A-infected HepG2 cells were transfected with the indicated siRNAs. After 24 and 48 hours, the nuclei (Nuc) were isolated. Co-immunoprecipitation assay was used to detect the interaction between ASPP2, p53, p-AKT and Bcl-2 in isolated nuclei. **F.** ChIP assay using an anti-p53 antibody to the p53-binding region in the *PUMA* promoter in rAd-A-infected HepG2 cells with or without siRNA knockdown of AKT/Bcl-2 (left panel). rAd-A-infected HepG2 cells were transfected or co-transfected with the indicated siRNAs for 24, 48 and 72 hours. Luciferase activity of the *PUMA* promoter-reporter is shown (right panel). Values are the mean ± SEM of triplicates.

Immunoblot assay showed that the HRAS/PI3K/AKT pathway was activated in HepG2 cells infected by rAd-ASPP2 for 48 and 72 hours, as the levels of HRAS-GTP, p-PI3K and p-AKT were increased (Figure 2A). siRNA knockdown of HRAS, PI3K, and AKT was able to recover ASPP2-induced apoptosis at 48 hours (Figure 2B). Here, nuclear translocation of Bcl-2 and p-AKT increased after rAd-ASPP2 infection for 48 hours (Figure 2C). Bcl-2 knockdown led to the recovery of ASPP2-induced apoptosis at 48 and 72 hours (Figure 2D). A CO-IP assay showed that nuclear Bcl-2, p-AKT, ASPP2 and p53 were in a larger complex (Figure 2E). ChIP and luciferase assays showed that knockdown of AKT or Bcl-2 recovered the interaction between p53 and the *PUMA* promoter and restored PUMA expression at 48 and 72 hours (Figure 2F).

### Nuclear p-AKT facilitates the interaction between Bcl-2 and p53, which releases p53 from the promoter region of pro-apoptotic genes

**Figure 3:**
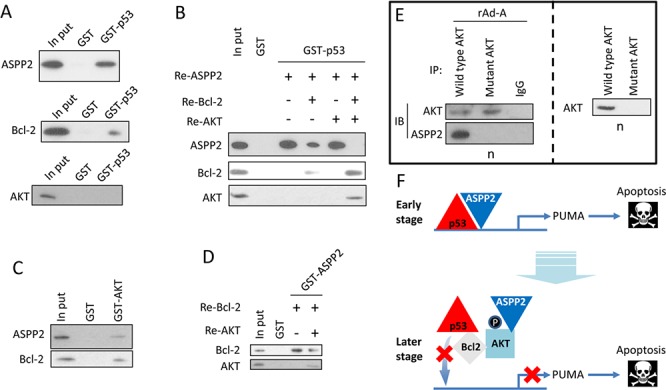
p-AKT facilitates the interaction between Bcl-2 and p53 in the nucleus **A.** Recombinant ASPP2 fragment (Re-ASPP2, which contains ankyrin repeats and SH3 domain, upper panel), Recombinant Bcl-2 (Re-Bcl-2, middle panel) or Recombinant AKT (Re-AKT, lower panel) was cultured with GST-p53. Immunoblot assay was used to detect the ASPP2-p53 (upper panel), p53-Bcl-2 (middle panel) or p53-AKT (lower panel) complex. **B.** Re-ASPP2, Re-Bcl-2, or Re-AKT was co-cultured with GST-p53. Immunoblot was used to detect the ASPP2-p53, p53-Bcl-2 or p53-AKT complex. **C.** Re-ASPP2 or Re-Bcl-2 was cultured with GST-AKT, and immunoblotting was used to detect the ASPP2-AKT or Bcl-2-AKT complex. **D.** Re-Bcl-2 and Re-AKT were co-cultured with GST-ASPP2 (which contains ankyrin repeats and SH3 domain), and immunoblotting was used to detect the ASPP2-AKT or ASPP2-Bcl-2 complex. **E.** rAd-ASPP2 (rAd-A)-infected HepG2 cells were transfected with wild type AKT plasmid or AKT-K179A, T308A, or S473A-mutant plasmid. CO-IP was used to detect the complex of ASPP2-wild type AKT or ASPP2-mutant AKT (right panel). Immunoblotting was used to detect the level of wild type AKT and mutant AKT in isolated nuclei (left panel). **F.** A model describes how ASPP2, AKT, Bcl-2, and p53 interact with each other in the nuclei.

The GST pull-down assay showed that p53 directly interacted with ASPP2 and Bcl-2, but not AKT (Figure 3A). Simultaneous culture of the recombinant ASPP2 fragment (Re-ASPP2), Bcl-2 (Re-Bcl-2) and GST-p53 showed that Bcl-2 competitively bound to p53, which suppressed the formation of the ASPP2-p53 complex (Figure 3B). Interestingly, after co-culture of Re-ASPP2, Re-Bcl-2, recombinant AKT (Re-AKT) and GST-p53, the ASPP2-p53 complex could not be detected, but the amount of p53-Bcl-2 complex was increased, suggesting that AKT facilitates the interaction between Bcl-2 and p53 (Figure 3B). Moreover, AKT directly bound to ASPP2 and Bcl-2 and decreased the formation of the ASPP2-Bcl-2 complex (Figures 3C and 3D). The overexpressed mutant form of AKT (K179A, T308A, S473A), which lacks the phosphorylation site, failed to be translocated to nucleus, and nuclear AKT-ASPP2 complex could not be detected, suggesting that phosphorylation of AKT is critical for its nuclear translocation (Figure 3E). The p53 DNA binding domain (p53DBD) is the binding site for Bcl-2 [16, 19]. Our data suggest that nuclear p-AKT facilitates the binding of Bcl-2 to p53DBD, which releases p53 from the promoter region of *PUMA* (Figure 3F).

### SOS1 activates the HRAS/PI3K/AKT pathway and is critical for nuclear translocation of p-AKT and Bcl-2

**Figure 4:**
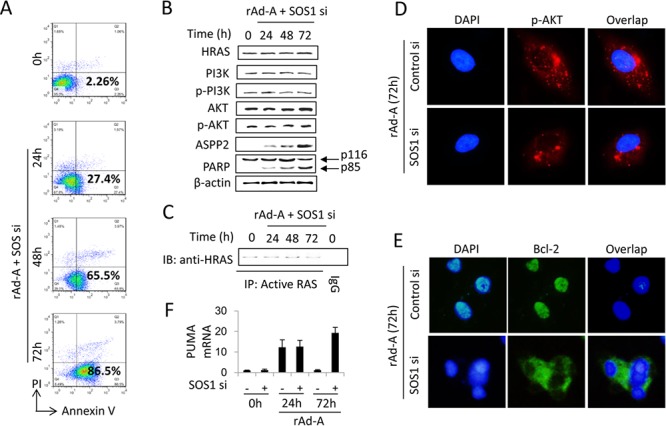
SOS1 expression is critical for activating the HRAS/PI3K/AKT pathway and maintaining nuclear translocation of p-AKT and Bcl-2 HepG2 cells were transfected with SOS1 siRNA (SOS1 si) for 24 hours and then infected by rAd-ASPP2 (rAd-A) for 72 hours. **A.** Annexin V/PI assay was used to detect ASPP2-induced apoptotic cells (Annexin V^+^ cells). **B** and **C.** The indicated antibodies were used to detect the HRAS/PI3K/AKT pathway (B); anti-RAS-GTP antibody was used to immunoprecipitate total activated RAS, and then anti-HRAS was used to detect activated HRAS (C). **D** and **E.**) Immunofluorescence assay was used to detect nuclear p-AKT (D, red) and Bcl-2 (E, green). DAPI was used to stain nuclei. **F.** Real-time PCR was used to detect PUMA mRNA. Values are the mean ± SEM of triplicates.

ASPP2 overexpression increased activated-HRAS levels (Figure 2A). HRAS is inactivated and activated by GTPase-activating proteins (GAPs) and guanine nucleotide exchange factors (GEFs), respectively [20, 21]. We employed real-time PCR to detect 40 GAP and GEF genes and determined that the mRNA levels of SOS1, RasGRP3, Ect2, and Collybistin were increased in HepG2 cells after rAd-ASPP2 infection for 48 and 72 hours (Figure S3A). The protein levels of SOS1 and Ect2 were also increased (Figure S3B). TUNEL assay showed that only siRNA knockdown of SOS1 was able to recover ASPP2-induced apoptosis at 48 and 72 hours (Figures S3C). Flow cytometry and immunoblot assays further showed that SOS1 knockdown recovered ASPP2-induced apoptosis by inactivating the HRAS/PI3K/AKT pathway (Figure 4A, 4B and 4C). SOS1 knockdown also inhibited nuclear translocation of p-AKT and Bcl-2 (Figure 4D and 4E) and recovered PUMA expression at 72 hours (Figure 4F), suggesting that SOS1 inhibits the binding of ASPP2-p53 complex to the promoter of *PUMA* by inducing nuclear translocation of p-AKT and Bcl-2.

### Nuclear EGFR promotes SOS1 expression by binding to its promoter

**Figure 5:**
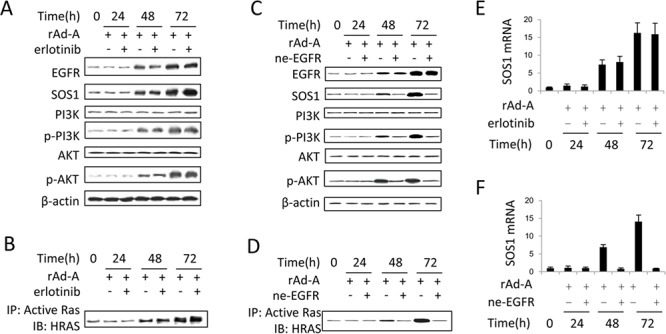
EGFR tyrosine kinase has no effect on activation of the SOS1/HRAS/PI3K/AKT pathway **A** and **C.** Immunoblot assay was used to detect the activation of the EGFR/SOS1/HRAS/PI3K/AKT pathway in rAd-ASPP2 (rAd-A)-infected HepG2 cells treated with erlotinib (A) or anti-EGFR neutralizing antibody (ne-EGFR) (C). **B** and **D.** Anti-RAS-GTP antibody was first used to immunoprecipitate total activated RAS, and then anti-HRAS antibody was used to detect activated HRAS in rAd-A-infected HepG2 cells treated with erlotinib (B) or ne-EGFR (D). **E** and **F.** Real-time PCR was used to detect the SOS1 levels. Values are the mean ± SEM of triplicates.

**Figure 6:**
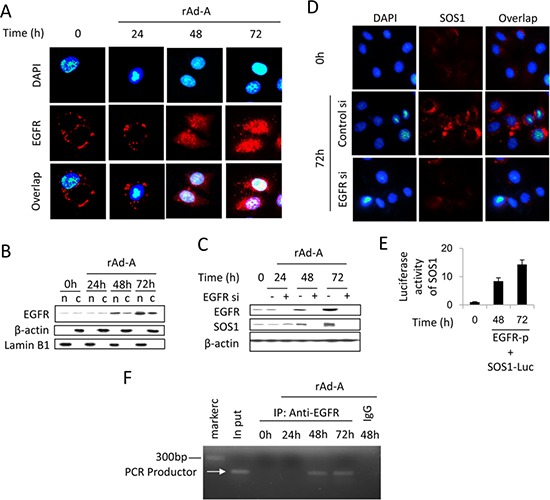
Nuclear EGFR positively regulates SOS1 expression **A.** Immunofluorescence was used to detect EGFR expression (red) in HepG2 cells after rAd-ASPP2 (rAd-A) infection for 24, 48 and 72 hours. Nuclei were stained with DAPI. **B.** HepG2 cells were infected with rAd-A for 24, 48 and 72 hours. Nuclei (n) and nuclei-free cytoplasm (c) were isolated, and immunoblot assay was used to detect the level of nuclear and cytoplasmic EGFR. **C.** HepG2 cells were infected with rAd-ASPP2 (rAd-A) with or without EGFR siRNA (EGFR si) treatment. Immunoblotting was used to detect EGFR and SOS1 expression. **D.** Immunofluorescence assay was used to detect SOS1 expression (red) in rAd-A-treated HepG2 cells for 72 hours with or without EGFR knockdown. Nuclei were stained with DAPI. **E.** Luciferase activity of SOS1. HepG2 cell were co-transfected with SOS1 reporter (SOS1-Luc) and EGFR (EGFR-p) plasmids for 48 and 72 hours. Values are the mean ± SEM of triplicates. **F.** Anti-EGFR antibody and control IgG were used for the chromatin immunoprecipitation (ChIP) assay in rAd-A-infected HepG2 cells, followed by PCR and ethidium bromide stained agarose gel electrophoresis.

ASPP2 overexpression enhanced EGF and EGFR expression at 48 and 72 hours (Figure S2A and S2B), and knockdown of EGF or EGFR recovered ASPP2-induced apoptosis (Figure S2D). The binding of EGF to EGFR also activates HRAS [21]. Erlotinib-induced inhibition of EGFR tyrosine kinase activity did not inactivate the HRAS/PI3K/AKT pathway and did not change SOS1 expression (Figure 5A, 5B and 5E). The binding of EGF to EGFR can induce nuclear translocation of EGFR [22]. An ne-EGFR was used to block the interaction between EGF and EGFR. ne-EGFR treatment inactivated the HRAS/PI3K/AKT pathway and decreased SOS1 expression (Figure 5C, 5D and 5F), indicating that nuclear EGFR might be required to induce SOS1 expression. Immunoblot and IF assays showed that nuclear translocation of EGFR was truly increased at 48 and 72 hours (Figure 6A and 6B), and ne-EGFR treatment decreased nuclear EGFR (Figure S4A). Real-time PCR, IF and immunoblot assays further showed that siRNA-mediated EGFR knockdown decreased SOS1 mRNA and protein levels (Figures 6C, 6D and S4B). EGFR overexpression increased SOS1 luciferase activity (Figure 6E). Finally, a ChIP assay demonstrated that nuclear EGFR bound to the *SOS1* promoter at 48 and 72 hours (Figure 6F). Thus, nuclear EGFR promotes SOS1 expression by directly binding to the promoter region of *SOS1*, which leads to SOS1-mediated activation of the HRAS/PI3K/AKT pathway.

### ne-EGFR improves and enhances ASPP2-induced apoptotic cell death *in vitro* and *in vivo*

**Figure 7:**
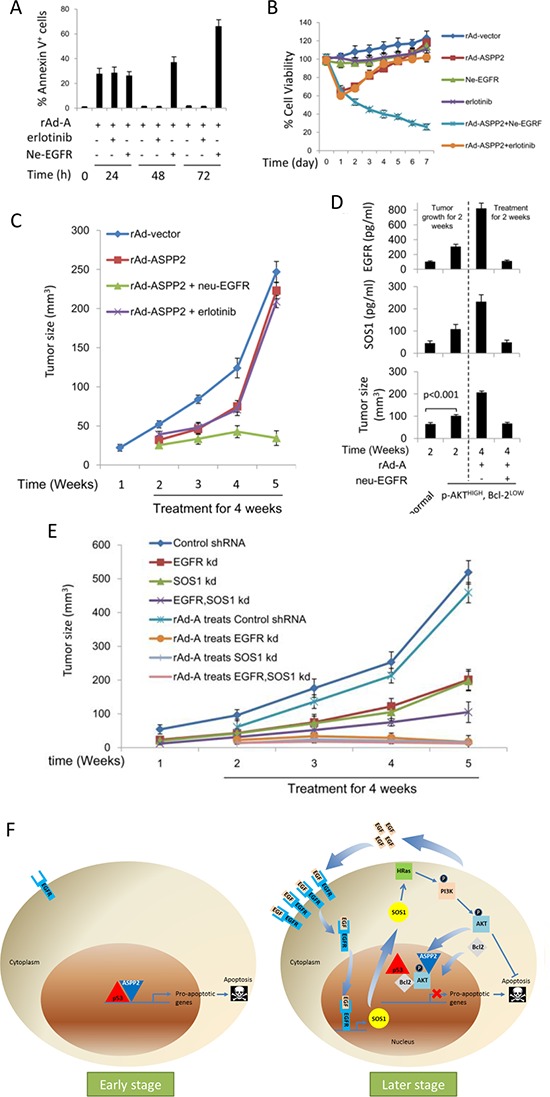
Anti-EGFR neutralizing antibody improves and enhances ASPP2-induced apoptosis *in vitro* and *in vivo* **A.** HepG2 cells were infected with rAd-ASPP2 (rAd-A) with or without co-treatment with erlotinib or anti-EGFR neutralizing antibody (ne-EGFR) for 24, 48 and 72 hours. Annexin V/PI assay was used to detect apoptosis (Annexin V^+^ cells). Values are the mean ± SEM of triplicates. **B.** MTT assay was used to detect cell viability after treatment with rAd-vector, rAd-ASPP2, ne-EGFR, erlotinib, rAd-ASPP2 combined with ne-EGFR and rAd-ASPP2 combined with erlotinib for 7 days in HepG2 cells. **C.** HepG2 cells were implanted subcutaneously in nude mice. After 1 week, mice were treated with rAd-vector, rAd-ASPP2, rAd-ASPP2 combined with ne-EGFR, and rAd-ASPP2 combined with erlotinib every week for a total of 4 times. At the indicated time points, tumor size was analyzed to evaluate the effect of the different treatments on inhibiting transplanted tumor growth *in vivo*. **D.** HepG2 cells were infected with rAd-ASPP2 for 72 hours. Flow cytometry was used to isolate p-AKT^HIGH^,Bcl-2^LOW^ cells. p-AKT^HIGH^, Bcl-2^LOW^ cells were implanted subcutaneously in nude mice, and normal HepG2 cells were used as a control. After 2 weeks, mice were treated with rAd-A and rAd-A combined with ne-EGFR every week, for a total of 2 times. After 2 weeks of treatment, tumor size was analyzed to evaluate the treatments (lower panel). ELISA was used to detect EGFR (upper panel) and SOS1 (middle panel) levels in the transplanted tumor. Values are the mean ± SEM of triplicates. **E.** EGFR knockdown (EGFR kd), SOS1 knockdown (SOS1 kd), EGFR and SOS1 dual knockdown (EGFR, SOS1 kd) HepG2 cell lines were implanted subcutaneously in nude mice for 1 week, after which rAd-A treatment was administered every week for a total of 4 times. At the indicated time points, tumor size was analyzed to evaluate the effect of rAd-A on treating transplanted tumor growth. Control shRNA treated-HepG2 cells were used as the control. **F.** A model to demonstrate how apoptosis is induced at early stages of ASPP2 overexpression and how ASPP2 overexpression fails to induce apoptosis at later stages.

The results of immunoblotting, flow cytometry and MTT assays showed that rAd-ASPP2 combined with ne-EGFR, but not erlotinib, effectively induced apoptotic cell death in HepG2 *in vitro* (Figure S5A, S5B, 7A and 7B). Nude mice were injected subcutaneously with HepG2 cells. EGFR levels were increased in the transplanted tumor treated with rAd-ASPP2 (Figure S5C). rAd-ASPP2 treatment significantly inhibited tumor growth, but treatment with rAd-ASPP2 combined with ne-EGFR was a more powerful inhibitor of transplanted tumor growth than rAd-ASPP2 treatment alone or rAd-ASPP2 combined with erlotinib (Figure 7C) because ne-EGFR extended and amplified ASPP2-induced apoptosis (Figure S5D).

It has been shown that ASPP2 overexpression reduces Bcl-2 levels, but the remaining Bcl-2 translocates to the nucleus [9]. p-AKT^HIGH^, Bcl-2^LOW^ cells (representing nuclear p-AKT^+^, Bcl-2^+^ cells) were isolated and used for subcutaneous injection for 2 weeks. p-AKT^HIGH^, Bcl-2^LOW^ cells grew faster than wild-type HepG2 cells (Figure 7D). EGFR and SOS1 levels were found to be higher in the p-AKT^HIGH^, Bcl-2^LOW^ HepG2-transplanted tumor than in the wild type HepG2 tumor (Figure 7D). rAd-ASPP2 combined with ne-EGFR, but not rAd-ASPP2 treatment alone, inhibited the growth of p-AKT^HIGH^, Bcl-2^LOW^ cells (Figure 7D). EGFR (EGFR kd), SOS1 (SOS1 kd) and dual protein (EGFR, SOS1 kd) knockdown cell lines were injected into nude mice. The three knockdown cells grew slowly, and rAd-ASPP2 treatment almost completely inhibited their growth (Figure 7E). Thus, EGFR/SOS1-promoted the growth of nuclear p-AKT^+^, Bcl-2^+^ cells results in the resistance of hepatoma cells to ASPP2-p53 complex-induced apoptosis.

### Activation of the HRAS/PI3K/AKT pathway by nuclear EGFR-induced SOS1 also impairs the pro-apoptotic function of cisplatin in HepG2 cells

To detect whether the activation of the SOS1/HRAS/PI3K/AKT pathway could impair other pro-apoptotic signals, we treated HepG2 cells with cisplatin. Apoptosis was detected at 24 and 48 hours using TUNEL assay, but the level of apoptosis at 48 hours was significantly decreased compared to that observed at 24 hours (Supplementary Figure 6A). Cisplatin treatment also increased the expression of EGF, EGFR and SOS1 and activated the HRAS/PI3K/AKT pathway at 48 hours, while knockdown of HRAS, PI3K or AKT enhanced cisplatin-induced apoptosis at 48 hours (Supplementary Figure 6A, 6B). The CO-IP assay showed that the level of ASPP2-p53 complex at 48 hours was the same as that at 24 hours (Supplementary Figure 6C). However, there was less p53 binding to the *PUMA* promoter and less PUMA expression at 48 hours compared to 24 hours, and knocking down HRAS, PI3K or AKT promoted the interaction between p53 and the *PUMA* promoter and increased PUMA expression, suggesting that p53 is released from the *PUMA* promoter at 48 hours in a HRAS/PI3K/AKT pathway-dependent manner (Supplementary Figure 6D).

Cisplatin treatment also induced nuclear translocation of EGFR, p-AKT and Bcl-2 at 48 hours (Supplementary Figure 7A). siRNA knockdown of EGFR, SOS1 or Bcl-2 enhanced cisplatin-induced apoptosis, PUMA expression and the binding of p53 to the *PUMA* promoter at 48 hours (Supplementary Figure 7B). Cisplatin treatment also induced the binding of EGFR to *SOS1* promoter at 48 hours (Supplementary Figure 7C). SOS1 mRNA and protein levels significantly increased at 48 hours, and EGFR knockdown reduced SOS1 mRNA levels, suggesting that EGFR directly induces SOS1 expression at 48 hours in cisplatin-treated HepG2 cells (Supplementary Figure 6B and 7D). Thus, activation of the HRAS/PI3K/AKT pathway by nuclear EGFR-induced SOS1 might be a general apoptosis-evasion mechanism in HCC in response to pro-apoptotic stimuli.

## DISCUSSION

Induction of p53-mediated apoptosis can be used to treat tumors (including HCC). Because ASPP2 has been reported to enhance the transactivation activity of p53 and subsequently the expression of pro-apoptotic genes, ASPP2 overexpression could be used to treat tumors by enhancing p53 activity [8]. Our study confirms that ASPP2 overexpression induces apoptotic cell death in HepG2 and PHCs by enhancing p53 activity. Two principle apoptosis signaling pathways have been delineated: extrinsic (death receptor) pathway and intrinsic (mitochondrial) pathway [1]. In this study, DR5 (representing the extrinsic pathway) had little effect on ASPP2-induced apoptosis, but PUMA, BAX and caspase 9 (representing the intrinsic pathway) were the main pro-apoptotic factors involved in ASPP2-induced apoptosis.

However, long-term ASPP2 overexpression (more than 48 hours) fails to induce apoptosis in HepG2 and PHCs, suggesting that hepatoma cells can also prevent long-term apoptotic stimuli. We believe that the plausible molecular mechanism in our setting is as follows: after long-term ASPP2 overexpression, the expression of EGF and EGFR is induced and EGFR is translocated to the nucleus by interaction with EGF. In the nucleus, EGFR induces SOS1 expression by directly binding to the *SOS1* promoter. SOS1 then activates the HRAS/PI3K/AKT pathway, which results in the nuclear translocation of p-AKT and Bcl-2. In the nucleus, the interaction between p-AKT and ASPP2 facilitates the direct interaction of Bcl-2 and p53, which impairs the formation of the ASPP2-p53 complex and then releases p53 from the pro-apoptotic gene promoters. Because p53 cannot bind to pro-apoptotic gene promoters, ASPP2 overexpression-induced apoptosis is blocked (Figure 7F). In addition, we also observed the same mechanism by which HepG2 cells are resistant to cisplatin treatment, suggesting that this new mechanism might be a common mechanism by which hepatoma cells produce an anti-apoptotic response when faced with pro-apoptotic signals.

Previous studies have reported that mitochondrial Bcl-2 plays an anti-apoptotic role by inhibiting p53-induced permeabilization of the mitochondrial outer membrane through direct binding with p53 [23]. We have previously demonstrated that ASPP2 overexpression reduces Bcl-2 levels by an unknown mechanism [9]. Here, we further determine that the presence of nuclear p-AKT is critical for nuclear Bcl-2 to inhibit apoptosis by blocking the interaction between p53 and the pro-apoptotic gene promoters via the Bcl-2-p53 interaction. The p53 DNA binding domain is the binding interface for Bcl-2 [16], and therefore, Bcl-2 can block p53 transactivation activity. Our data are consistent with the conclusion that the ratio of anti-apoptotic *versus* pro-apoptotic Bcl-2 proteins, rather than the expression levels of one particular molecular of the Bcl-2 family, regulates apoptosis sensitivity [2].

The nuclear translocation of p-AKT and Bcl-2 is directly responsible for the inhibition of ASPP2-induced apoptosis. Recombinant ASPP2 contains ankyrin repeats and SH3 domains, which are required for its specific interaction with p53 [5]. The proline-rich region in the C-terminus of AKT binds SH3 domain-containing proteins, explaining why AKT directly binds ASPP2 [24]. Moreover, the ankyrin repeats and SH3 domains in the C-terminus of ASPP2 not only bind p53 but are also required to bind Bcl-2 [25]. Our data show that although Bcl-2 cannot completely block the interaction between ASPP2 and p53, in the presence of AKT, Bcl-2 almost completely blocks the interaction between ASPP2 and p53. We believe that the presence of AKT facilitates the interaction between Bcl-2 and p53.

In our setting, EGFR-induced SOS1 expression is critical for activating the HRA/PI3K/AKT pathway and maintaining nuclear translocation of p-AKT and Bcl-2. EGFR tyrosine kinase activity is not critical for the survival of non-apoptotic cells, but the nuclear translocation of EGFR is required for producing the anti-apoptotic response. Nuclear EGFR binds to the promoter of *SOS1* and induces SOS1 expression. SOS1 activates HRAS and then activates the PI3K/AKT pathway. To our knowledge, this is the first study to identify that EGFR is involved in directly regulating SOS1 expression.

Although sorafenib has been demonstrated to significantly improve the overall survival of patients with advanced HCC, alternative and more active agents targeting novel signaling pathways are still necessary for improving systemic therapy in HCC [26]. One of the hallmarks of tumors is the intrinsic or acquired resistance to apoptosis. Evasion of apoptosis may contribute to carcinogenesis, tumor progression and treatment resistance [2]. Some apoptosis mediators help determine whether cells undergo successful apoptosis or an unsuccessful one, and they sometimes act as targets for drug discovery [27]. Although EGFR blockage can enhance apoptosis susceptibility in tumor cells [28], our data demonstrate that blocking EGFR tyrosine kinase by erlotinib fails to recover ASPP2 overexpression-induced apoptosis in HCC. A previous study also reported that adding erlotinib to sorafenib does not improve survival in patients with advanced HCC [29], suggesting that EGFR signaling is not the right pathway to target [26]. However, our data that nuclear translocation of EGFR plays an anti-apoptotic role in HCC suggest that blocking nuclear translocation of EGFR might be an alternative way to treat HCC. To our knowledge, SOS1 has rarely been the focus in HCC research. In our study, SOS1 expression appears to be involved in nuclear EGFR-mediated apoptosis evasion. Recently, EGFR has been reported to use SOS1 to drive constitutive activation of Nuclear Factor κB (NFκB) in cancer cells [30]. NFκB is abnormally constitutively activated in most cancers (including HCC) and it always plays a role for promoting resistance to apoptosis and contributing to tumorigenesis [30, 31]. Thus, SOS1 is an important downstream protein of EGFR, and it is rational to develop a SOS1 inhibitor for HCC treatment. This is supported by the fact that our *in vivo* and *in vitro* results demonstrate that blocking nuclear translocation of EGFR dramatically improves the pro-apoptotic function of ASPP2 by reducing SOS1 expression.

Taken together, we believe that our data could explain some of the cases where p53 is normal but apoptosis cannot be successfully induced. Furthermore, our data suggest that rAd-ASPP2 combined with ne-EGFR might be used to treat HCC effectively.

## MATERIALS AND METHODS

Cell culture of HepG2 and human primary hepatoma cells (PHCs), transfection of plasmids and siRNAs, cisplatin treatment, immunoblotting, real-time PCR and chromatin immunoprecipitation (ChIP) assays were performed as previously described [9, 18], The effects of recombinant adenovirus-ASPP2 (rAd-ASPP2), rAd-ASPP2 combined with anti-EGFR neutralizing antibody (ne-EGFR) or erlotinib on inhibiting tumor growth were detected in HepG2-transplanted xenograft tumors. For more detailed methods, see Supplementary Materials and Methods.

## SUPPLEMENTARY DATA FIGURES



## ABBREVIATIONS

ASPP2: Apoptosis-stimulating protein of p53-2
rAd-A: recombinant adenovirus-ASPP2
p53: tumor protein p53
PARP: poly (ADP-ribose) polymerase 1
EGF: epidermal growth factor
EGFR: epidermal growth factor receptor
SOS1: Son of Sevenless 1
HRAS: Harvey rat sarcoma viral oncogene homolog
PI3K: phosphatidylinositol 3-kinase
PUMA: p53 upregulated modulator of apoptosis
AKT: v-akt murine thymoma viral oncogene homolog 1
Bcl-2: B-cell CLL/lymphoma 2
DR5: death receptor 5
APAF1: apoptotic peptidase activating factor 1
BAX: BCL2-associated X protein
FAS: Fas cell surface death receptor
EI24: etoposide induced 2.4
TRAIL: TNF-related apoptosis-inducing ligand
HB-EGF: heparin-binding EGF-like growth factor
TGFA: transforming growth factor
RasGRP3: RAS guanyl releasing protein 3
Ect2: epithelial cell transforming 2
Collybistin: Cdc42 guanine nucleotide exchange factor (GEF) 9
DAPI: 4′,6-diamidino-2-phenylindole
IF assay: Immunofluorescence assay
HCC: Hepatocellular carcinoma

## References

[1] Hengartner MO (2000). The biochemistry of apoptosis. Nature.

[2] Fulda S (2009). Tumor resistance to apoptosis. Int J Cancer.

[3] El-Serag HB, Rudolph KL (2007). Hepatocellular carcinoma: epidemiology and molecular carcinogenesis. Gastroenterology.

[4] Penuelas I, Mazzolini G, Boan JF, Sangro B, Marti-Climent J, Ruiz M (2005). Positron emission tomography imaging of adenoviral-mediated transgene expression in liver cancer patients. Gastroenterology.

[5] Bergamaschi D, Samuels Y, O'Neil NJ, Trigiante G, Crook T, Hsieh JK (2003). iASPP oncoprotein is a key inhibitor of p53 conserved from worm to human. Nat Genet.

[6] Samuels-Lev Y, O'Connor DJ, Bergamaschi D, Trigiante G, Hsieh JK, Zhong S (2001). ASPP proteins specifically stimulate the apoptotic function of p53. Mol Cell.

[7] Bergamaschi D, Samuels Y, Jin B, Duraisingham S, Crook T, Lu X (2004). ASPP1 and ASPP2: common activators of p53 family members. Mol Cell Biol.

[8] Kampa KM, Acoba JD, Chen D, Gay J, Lee H, Beemer K (2009). Apoptosis-stimulating protein of p53 (ASPP2) heterozygous mice are tumor-prone and have attenuated cellular damage-response thresholds. Proc Natl Acad Sci U S A.

[9] Liu K, Shi Y, Guo X, Wang S, Ouyang Y, Hao M (2014). CHOP mediates ASPP2-induced autophagic apoptosis in hepatoma cells by releasing Beclin-1 from Bcl-2 and inducing nuclear translocation of Bcl-2. Cell Death Dis.

[10] Nakanishi K, Sakamoto M, Yamasaki S, Todo S, Hirohashi S (2005). Akt phosphorylation is a risk factor for early disease recurrence and poor prognosis in hepatocellular carcinoma. Cancer.

[11] Sarbassov DD, Guertin DA, Ali SM, Sabatini DM (2005). Phosphorylation and regulation of Akt/PKB by the rictor-mTOR complex. Science.

[12] Schmitz KJ, Wohlschlaeger J, Lang H, Sotiropoulos GC, Malago M, Steveling K (2008). Activation of the ERK and AKT signalling pathway predicts poor prognosis in hepatocellular carcinoma and ERK activation in cancer tissue is associated with hepatitis C virus infection. J Hepatol.

[13] Cully M, You H, Levine AJ, Mak TW (2006). Beyond PTEN mutations: the PI3K pathway as an integrator of multiple inputs during tumorigenesis. Nat Rev Cancer.

[14] Hemann MT, Lowe SW (2006). The p53-Bcl-2 connection. Cell Death Differ.

[15] Petros AM, Gunasekera A, Xu N, Olejniczak ET, Fesik SW (2004). Defining the p53 DNA-binding domain/Bcl-x(L)-binding interface using NMR. Febs Lett.

[16] Tomita Y, Marchenko N, Erster S, Nemajerova A, Dehner A, Klein C (2006). WT p53, but not tumor-derived mutants, bind to Bcl2 via the DNA binding domain and induce mitochondrial permeabilization. J Biol Chem.

[17] Froesch BA, Aime-Sempe C, Leber B, Andrews D, Reed JC (1999). Inhibition of p53 transcriptional activity by Bcl-2 requires its membrane-anchoring domain. J Biol Chem.

[18] Taira N, Yamaguchi T, Kimura J, Lu ZG, Fukuda S, Higashiyama S (2014). Induction of amphiregulin by p53 promotes apoptosis via control of microRNA biogenesis in response to DNA damage. Proc Natl Acad Sci U S A.

[19] Sot B, Freund SM, Fersht AR (2007). Comparative biophysical characterization of p53 with the pro-apoptotic BAK and the anti-apoptotic BCL-xL. J Biol Chem.

[20] Schmidt A, Hall A (2002). Guanine nucleotide exchange factors for Rho GTPases: turning on the switch. Genes Dev.

[21] Ksionda O, Limnander A, Roose JP (2013). RasGRP Ras guanine nucleotide exchange factors in cancer. Front Biol (Beijing).

[22] Brand TM, Iida M, Li C, Wheeler DL (2011). The nuclear epidermal growth factor receptor signaling network and its role in cancer. Discov Med.

[23] Jiang M, Milner J (2003). Bcl-2 constitutively suppresses p53-dependent apoptosis in colorectal cancer cells. Genes Dev.

[24] Du K, Tsichlis PN (2005). Regulation of the Akt kinase by interacting proteins. Oncogene.

[25] Naumovski L, Cleary ML (1996). The p53-binding protein 53BP2 also interacts with Bc12 and impedes cell cycle progression at G2/M. Mol Cell Biol.

[26] Wörns MA, Galle PR (2014). HCC therapies—lessons learned. Nat Rev Gastroenterol Hepatol.

[27] Hassan M, Watari H, AbuAlmaaty A, Ohba Y, Sakuragi N (2014). Apoptosis and molecular targeting therapy in cancer. Biomed Res Int.

[28] Kari C, Chan TO, Rocha de Quadros M, Rodeck U (2003). Targeting the epidermal growth factor receptor in cancer: apoptosis takes center stage. Cancer Res.

[29] Zhu AX, Rosmorduc O, Evans TR, Ross PJ, Santoro A, Carrilho FJ (2015). SEARCH: A Phase III, Randomized, Double-Blind, Placebo-Controlled Trial of Sorafenib Plus Erlotinib in Patients With Advanced Hepatocellular Carcinoma. J Clin Oncol.

[30] De S, Dermawan JK, Stark GR (2014). EGF receptor uses SOS1 to drive constitutive activation of NFκB in cancer cells. Proc Natl Acad Sci U S A.

[31] Perkins ND (2012). The diverse and complex roles of NF-kB subunits in cancer. Nat Rev Cancer.

