# Supplementary material for: Nuclear EGFR impairs ASPP2-p53 complex-induced apoptosis by inducing SOS1 expression in hepatocellular carcinoma
Source: Oncotarget. 2015 Apr 27;6(18):16507–16. doi: 10.18632/oncotarget.3757 (PMC4599285; doi:10.18632/oncotarget.3757)
Supplement: Supplementary file 1 [file oncotarget-06-16507-s001.pdf]

SUPPLEMENTARY DATA

Cell culture and treatment

HepG2 cells were grown in Dulbecco's modified Eagle's medium (DMEM). DMEM was supplemented with 10% fetal bovine serum (FBS). Human primary hepatoma cells (PHCs) were isolated from specimens obtained from patients undergoing hepatic resections. The specimens used in this study were obtained for pathological examination; therefore, there was no need for the patient to sign an informed consent form. PHCs were isolated from prewashed livers using a two-step collagenase perfusion and were cultivated within two layers of rat-tail collagen [1, 2]. All cells were seeded in 6- or 24-well plates and were transfected with ASPP2/Vector plasmid or treated by cisplatin. The siRNAs against HRAS, SOS1, p85 PI3K, EGFR and AKT were transfected by Fugene HD (Promega, Madison, WI). The siRNAs were purchased from C*ell Signaling Technology* and Novus Biologicals. The cells were grown on glass cover slips for the immunofluorescence studies.

In vivo study

HepG2 cells were harvested, washed and re-suspended in DMEM medium. Male nude mice (strain BALB/c nude; Beijing), 4–5 weeks old, were received subcutaneous injection of 5×10^5 HepG2 cells. After 1 week (time zero), mice were randomized into four groups: rAd-ASPP2, rAd-ASPP2 combined with neutralizing EGFR antibody, rAd-ASPP2 combined with erlotinib and control treated by rAd-vector. At time zero and time 1, 2, 3 weeks, rAd-ASPP2 and rAd-vector were administrated intratumorally in mice. At same time, mice were treated with intraperitoneal injection (i.p) of neutralizing EGFR antibody and erlotinib. Nuclear p-AKT^{HIGH}, Bcl-2^{LOW} cells were isolated by flow cytometry and were implanted subcutaneously in nude mice. After 2 weeks, mice were treated with rAd-ASPP2 and rAd-ASPP2 combined with ne-EGFR for 2 every week, total 2 times. After 2 weeks treatment, tumor size was analyzed for evaluation of the treatments. All animal work was done in accordance with protocol approved by the institutional guidelines committee of Beijing You'an Hospital.

Immunoblot assay

Cell lysates were subjected to immunoblot analysis, as previously described [3]. Briefly, total cellular lysates were separated on 10% or 15% SDS-PAGE gels, and the separated proteins were then transferred to PVDF membranes. The protein blots were blocked with 5% non-fat milk and sequentially probed with specific primary antibodies and horseradish peroxidase-conjugated secondary antibodies. The detection of specific proteins on the blots was achieved using an enhanced chemiluminescence system (Pierce SuperSignal, Thermo Fisher Scientific Inc. Rockford, IL), and the results were captured on X-ray film or ImageQuant LAS 4000. Densitometric analysis was performed using the Image-Pro Plus analysis software.

Real-time PCR

The RNeasy Mini Kit (Qiagen, Hilden, Germany) was used to isolate total RNA from the cultured cells. Reverse transcription was used to synthesize first strand cDNA using the SuperScript II First-Strand Synthesis System for RT-PCR (Invitrogen, Carlsbad, CA). SYBR Green was used to detect the dsDNA products during the real-time PCR reaction. The mRNA content was normalized to the expression of the housekeeping gene β-actin. The following specific primer sequences were used for real-time PCR: β-actin, 5′-GCCCTGAGGCACTCTTCCA-3′ (forward) and 5′-CGGATGTCCACGTCACACTT-3′ (reverse). The PUMA and SOS1 primers came from PrimePCR™ SYBR Green ® Assay (Bio-RAD).

Chromatin Immunoprecipitation (CHIP)

CHIP analysis was performed as described [4]. Briefly, The PCR of the AREG and SOS1 promoter sequence was performed using 35 cycles as follows: 94°C for 15 seconds, 65°C for 45 seconds. The resulting PCR products were analyzed on 1.5% TAE-agarose gels. The SOS1-specific primer sequences are as follows: forward, 5′ GATATCGAGACCTCTTTTAC 3′ and reverse, 5′ CCGGGACCACTTTTTCTAGA 3′. For PUMA promoter, forward, 5′ GCGAGACTGTGGCCTTGTGT 3′ and reverse, 5′ CGTTCCAGGGTCCACAAAGT 3′ [5].

Fluorescence microscopy

Frozen cells were fixed with 10% paraformaldehyde/PBS, incubated in 1% Triton X-100/PBS for 5 min, blocked with 3% BSA/PBS, and probed with EGRR antibodies. Nuclei were counterstained with 4',6-diamidino-2-phenylindole (DAPI). Immunofluorescence images were detected using a fluorescence microscope (Nikon Eclipse 80i). For quantitative analysis, at least 500 cells were counted for each sample.

Co-immunoprecipitation assay (CO-IP)

CO-IP was performed as described [3]. Cell lysates (1000 μg of protein in 500 μl of RIPA lysis buffer) were pre-cleared using protein A/G PLUS-agarose beads (Santa Cruz Biotechnology) and were incubated at 4°C overnight with anti-ASPP2, anti-HRas, anti-RAS-GTP, anti-p53, anti-Bcl-2, anti-p-AKT antibodies. The immunocomplexes were separated by incubation with protein A/G agarose beads and were resolved using SDS-PAGE. Western blot analysis was performed to detect the level of protein.

GST pull down assay

GST-tagged p53, AKT and ASPP2 fragment were incubated with recombinant ASPP2 fragment, Bcl-2 and AKT at 4°C for 1 hour and bound complex was pulled down by incubating with glutathione beads for 45 min followed by three washs with lysis buffer as described [6]. The amino acids sequence of GST-tagged ASPP2 fragment and recombinant ASPP2 fragment was from 512 to 611, and the sequence contains ankyrin repeats and SH3 domain of ASPP2 which are required for a specific interaction with p53.

Subcellular fractionation

Subcellular fractionation was performed as described [3]. Briefly, cells were Dounce homogenized on ice in M-SHE buffer [0.21 mol/L mannitol, 0.07 mol/L sucrose, 10 mmol/L HEPES-KOH (pH 7.4), 1 mmol/L EDTA, 1 mmol/L EGTA, 0.15 mmol/L spermine, 0.75 mmol/L spermidine, 1 mmol/L DTT] with freshly added protease inhibitors (1 μg/mL of leupeptin, aprotinin, pepstatin A, 1 mmol/L phenylmethylsulfonyl fluoride). Nuclei were pelleted at 1,200 × *g*, and the supernatant was taken as the cytoplasmic fraction.

Cell viability and apoptosis analysis

Cell viability was quantified with the MTT colorimetric assay kit (Promega), and cell apoptosis was detected by flow cytometric analysis following staining with Annexin V/PI (eBioscience), according to the instructions of the manufacturer. Apoptosis was also detected by Tunel assay (Promega) according to the instructions of the manufacturer.

Luciferase reporter assay

The detailed methods have been described previously [7]. Briefly, HepG2 cells were transfected with ASPP2 plasmid, EGFR plasmid, SOS1 report pasmid and PUMA reporter plasmid. Relative light unit values were normalized to β–galactosidase signal. One microgram of each indicated GPC3 promoter-reporter plasmid and pRSV β-galactosidase plasmid was used for all transfections.

Cluster assay

PCR array (QIAGEN) was used to detect the expression of liver cancer-related genes and p53 target genes (total 84 genes) in HepG2 cells infected by rAd-ASPP2 for 8, 16, 24, 48 and 72 hours. Time 0 was control group. The up- or down-regulation of each gene in 8, 16, 24, 48 and 72 hours was compared with time 0. The $2^{-\Delta\Delta C_T}$ value was used to represent up- or down-regulation. Cluster 3.0 was used to analyze $2^{-\Delta\Delta C_T}$ value of each gene in 8, 16, 24, 48 and 72 hours. Both genes and arrays were clustered. TreeView was used to produce heatmap.

Statistical analysis

All data shown are the results of at least three independent experiments and are expressed as the mean ± SEM. The differences between groups were compared using Student's *t*-test. Differences were considered significant at confidence levels of $p < 0.05$, $p < 0.01$ and $p < 0.001$, as indicated.

REFERENCES

1. Seglen PO. Preparation of isolated rat liver cells. Methods Cell Biol. 1976; 13:29–83.
2. Elsdale T, Bard J. Collagen substrata for studies on cell behavior. J Cell Biol. 1972; 54:626–637.
3. Liu K, Shi Y, Guo X, Wang S, Ouyang Y, Hao M, et al. CHOP mediates ASPP2-induced autophagic apoptosis in hepatoma cells by releasing Beclin-1 from Bcl-2 and inducing nuclear translocation of Bcl-2. Cell Death Dis. 2014; 5:e1323.
4. Li L, Jin R, Zhang X, Lv F, Liu L, Liu D, et al. Oncogenic activation of glypican-3 by c-Myc in human hepatocellular carcinoma. Hepatology. 2012; 56:1380–1390.
5. Nakano K, Vousden KH. PUMA, a novel proapoptotic gene, is induced by p53. Mol Cell. 2001; 7:683–694.
6. Wang Z, Liu Y, Takahashi M, Van Hook K, Kampa-Schittenhelm KM, Sheppard BC, et al. N terminus of ASPP2 binds to Ras and enhances Ras/Raf/MEK/ERK activation to promote oncogene-induced senescence. Proc Natl Acad Sci U S A. 2013; 110:312–317.
7. Chen D, Padiernos E, Ding F, Lossos IS, Lopez CD. Apoptosis-stimulating protein of p53-2 (ASPP2/53BP2L) is an E2F target gene. Cell Death Differ. 2005; 12:358–368.

SUPPLEMENTARY FIGURES

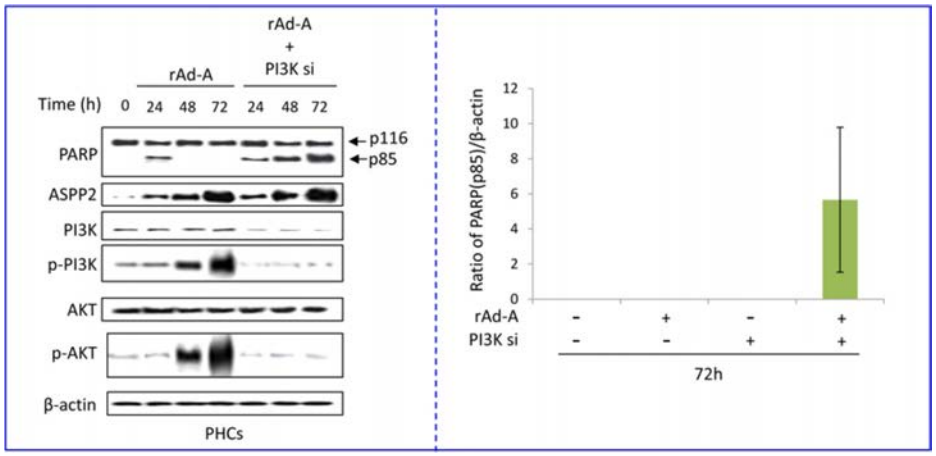

Supplementary Figure S1: ASPP2-induced apoptosis is impaired in human primary hepatoma cells (PHCs). Representative images of immunoblot of rAd-ASPP2 (rAd-A) or rAd-Vector (rAd-V) infected PHCs with or without PI3K inhibition via siRNA (left panel). Densitometry analysis of the band density ratio of PARP (p85) to β-actin in 7 HCC patients (right panel). Values are mean ± SEM of triplicates.

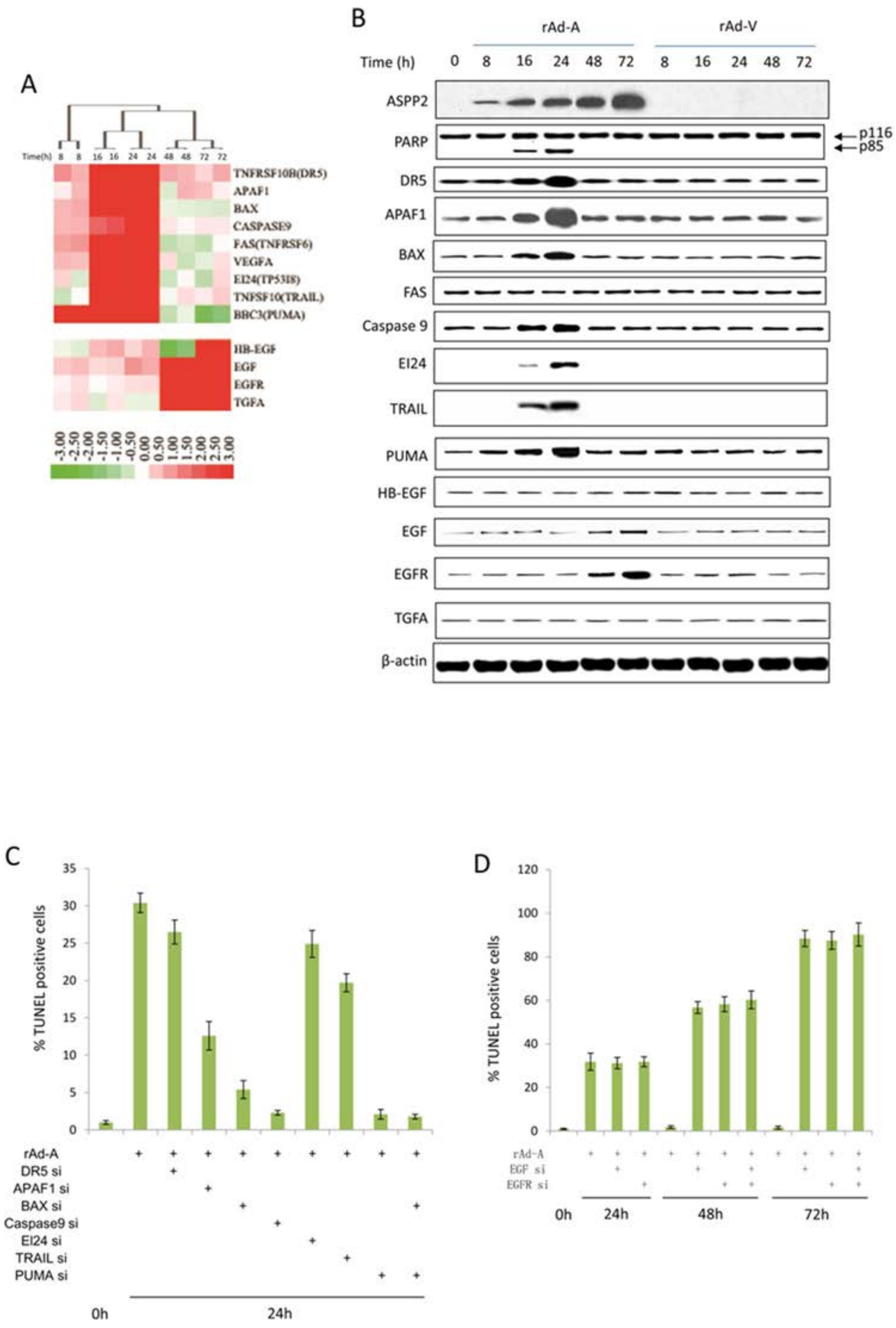

Supplementary Figure S2: PCR array was used to analyze the expression of liver cancer related genes and p53 target genes. A. Representative heatmap of p53 target genes and EGFR pathway-related genes in HepG2 infected by rAd-ASPP2 (rAd-A) for 8, 16, 24, 48 and 72 hours. HepG2 cells were infected by rAd-A for 8, 16, 24, 48 and 72 hours. Total RNA at time 0, 8, 16, 24, 48 and 72 was isolated and then was used to produce cDNA. PCR array was used to detect the expression of liver cancer-related genes and p53 target genes (total 84 genes). The up-regulation or down-regulation of each gene at 8, 16, 24, 48 and 72 hours is represented by $2^{-\Delta\Delta C_T}$ value. The $2^{-\Delta\Delta C_T}$ value of each gene at 8, 16, 24, 48 and 72 hours was analyzed by cluster 3.0 and heatmap is produced by TreeView. **B.** Immunoblot assay was used to detect the protein level of indicated genes at time 0, 8, 16, 24, 48 and 72 in HepG2 cells infected by rAd-A and rAd-Vector (rAd-V). **C** and **D.** Knockdown of indicated genes via siRNAs and Tunel was used to detect apoptotic cells in HepG2 cells infected by rAd-A.

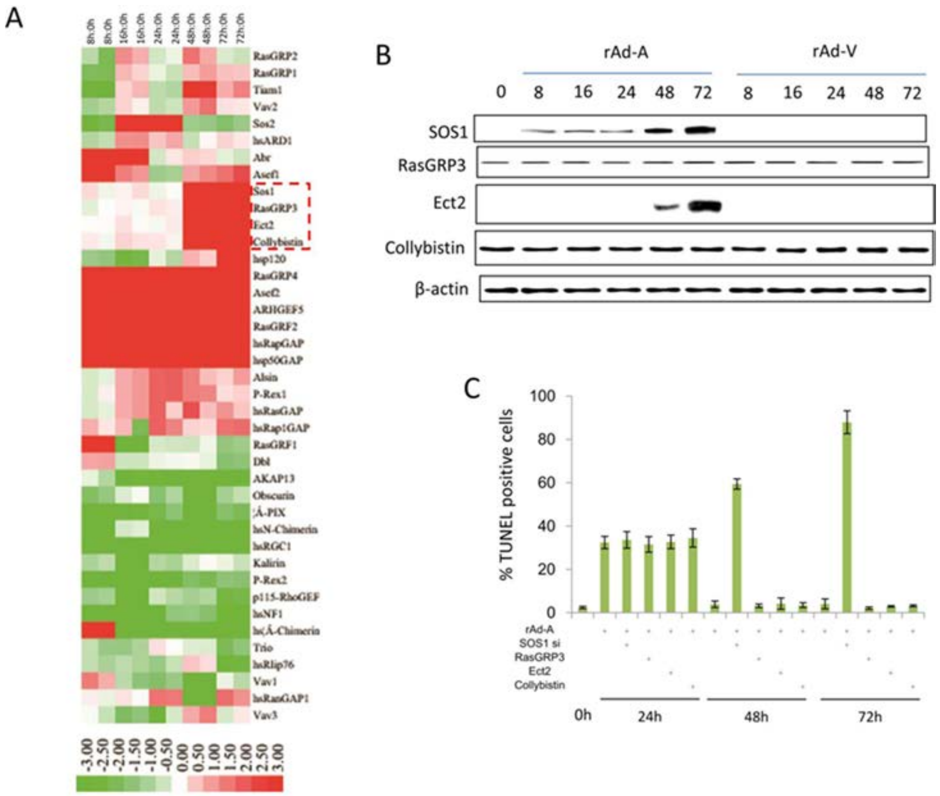

Supplementary Figure S3: Detection the expression of GEFs and GAPs-related genes. A. Heatmap was used to show the up- or down-regulation of genes in HepG2 infected by rAd-ASPP2 (rAd-A) for 8, 16, 24, 48 and 72 hours. Real time PCR and was used to detect the expression of 40 GEFs and GAPs-related genes in HepG2 cells infected by rAd-A for 8, 16, 24, 48, 72 hours. Cluster 3.0 was used to analyze $2^{-\Delta\Delta C_T}$ value of each genes at 8, 16, 24, 48, 72 hours and TreeView was used to produce heatmap. **B.** Immunoblot assay was used to detect the expression of indicated genes in HepG2 cells infected by rAd-A and rAd-Vector (rAd-V). **C.** Knockdown of indicated genes via siRNA and Tunel was used to detect apoptosis inHepG2 cells infected by rAd-A.

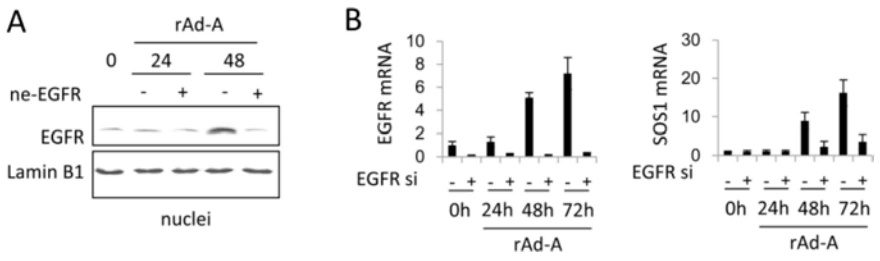

Supplementary Figure S4: Detection of nuclear EGFR and detection mRNA level of EGFR and SOS1. A. HepG2 cells were infected by rAd-ASPP2 (rAd-A) with or without anti-EGFR neutralizing antibody (ne-EGFR) treatment for 24 and 48 hours. Nuclei were isolated and immunoblot was used to detect nuclear EGFR level. **B.** HepG2 cells were infected by rAd-A with or without EGFR siRNA (EGFR si) treatment. Real time PCR was used to detect EGFR and SOS1 expression. Values are mean ± SEM of triplicates.

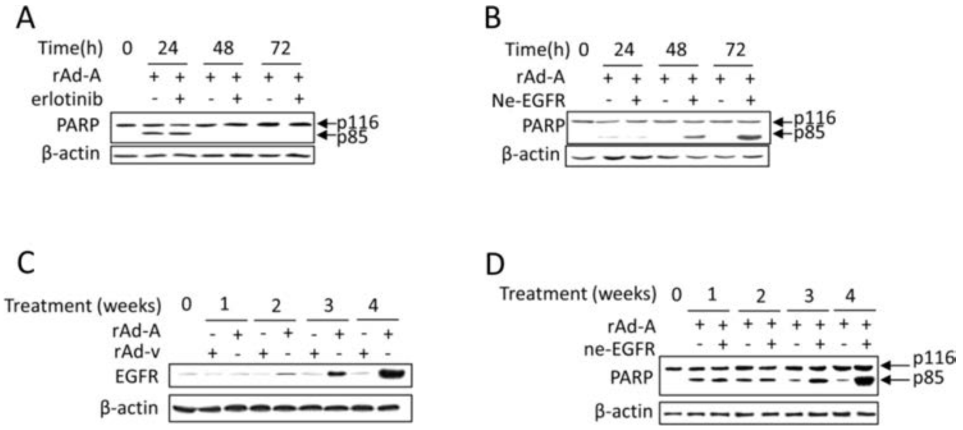

Supplementary Figure S5: Detection of apoptosis and EGFR by immunoblot. A and B. HepG2 cells were infected by rAd-ASPP2 (rAd-A) with or without co-treatment with erlotinib (A) or anti-EGFR neutralizing antibody (ne-EGFR) (B) for 24, 48 and 72 hours. Immunoblot was used to detect PARP. **C** and **D.** HepG2 cells were implanted subcutaneously in nude mice. After 1 week, mice were treated with rAd-vector, rAd-ASPP2, rAd-ASPP2 combined with ne-EGFR every week, total 4 times. Immunoblot assay was used to detect EGFR (C) and PARP (D).

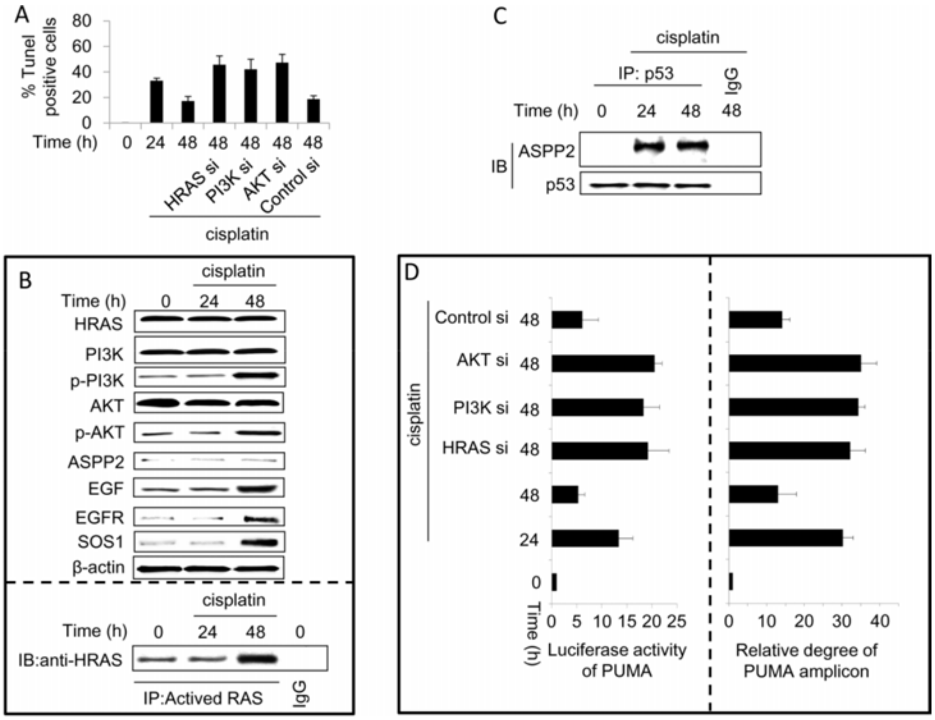

Supplementary Figure S6: Cisplatin treatment activates SOS1/HRAS/PI3K/AKT pathway in HepG2 cells. HepG2 cells were treated by cisplatin for 24 and 48 hours with or without pre-treatment by HRAS or PI3K or AKT siRNA. **A.** Tunel was used to detect apoptosis in HepG2 cells. Values are mean ± SEM of triplicates. (B, upper panel) Immunoblot assay was used to detect the activation of HRAS/PI3K/AKT pathway in HepG2 cell treated by cisplatin for indicated times. (B, lower panel) Anti-RAS-GTP antibody (activated RAS) was used to immunoprecipite total activated RAS, anti-HRAS was used to detect HRAS level in total activated RAS. **C.** CO-IP assay was used to detect the formation of ASPP2-p53 complex in HepG2 cells treated by cisplatin for indicated times. **D.** cisplatin-treated HepG2 cells were transfected with indicated siRNAs for 48 hours. Luciferase activity of PUMA promoter-reporter was shown (left panel); a ChIP assay using an anti-p53 antibody to the p53-binding region in *PUMA* promoter (right panel). Values are mean ± SEM of triplicates.

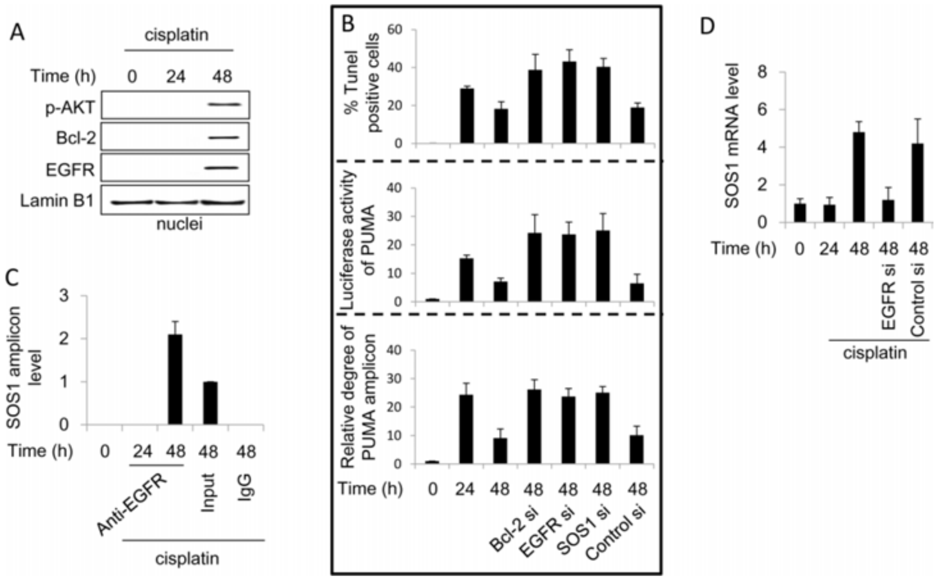

Supplementary Figure S7: Inhibiting EGFR/SOS1 via siRNA enhances cisplatin-induced apoptosis in HepG2 cells. A. HepG2 cells were treated by cisplatin for 24 and 48 hours. Immunoblot assay was used to detected nuclear p-AKT, Bcl-2 and EGFR in isolated nuclei by indicated antibodies. **B.** HepG2 cells were treated by cisplatin for 24 and 48 hours with or without pre-treatment by siRNA of Bcl-2 or EGFR or SOS1. Apoptosis was detected by Tunel assay (upper panel); middle panel showed the luciferase activity of *PUMA*; lower panel showed a ChIP assay using an anti-p53 antibody to the p53-binding region in *PUMA* promoter (right panel). Values are mean ± SEM of triplicates. **C.** A ChIP assay using an anti-EGFR antibody to the EGFR-binding region in *SOS1* promoter in cisplatin-treated HepG2 cells. Values are mean ± SEM of triplicates. **D.** Real time PCR was used to detect the level of SOS1 mRNA in HepG2 cell treated by cisplatin for indicated times with or without pre-treatment by EGFR/control siRNA. Values are mean ± SEM of triplicates.
